# Stem-Loop RT-qPCR as an Efficient Tool for the Detection and Quantification of Small RNAs in *Giardia lamblia*

**DOI:** 10.3390/genes7120131

**Published:** 2016-12-20

**Authors:** Jaime Marcial-Quino, Saúl Gómez-Manzo, Francisco Fierro, America Vanoye-Carlo, Yadira Rufino-González, Edgar Sierra-Palacios, Adriana Castillo-Villanueva, Rosa Angélica Castillo-Rodríguez, Eduardo Rodríguez-Bustamante, Roberto Arreguin-Espinosa, Horacio Reyes-Vivas

**Affiliations:** 1 CONACYT-Instituto Nacional de Pediatría, Secretaría de Salud, Ciudad de México 04530, Mexico; racastilloro@conacyt.mx; 2 Laboratorio de Bioquímica Genética, Instituto Nacional de Pediatría, Secretaría de Salud, Ciudad de México 04530, Mexico; saulmanzo@ciencias.unam.mx (S.G.-M.); acastilloinp@gmail.com (A.C.-V.); hreyesvivas@yahoo.com.mx (H.R.-V.); 3 Laboratorio de Ingeniería Genética y Metabolitos Secundarios, Departamento de Biotecnología, Universidad Autónoma Metropolitana-Iztapalapa, Ciudad de México 09340, Mexico; degfff@yahoo.com; 4 Laboratorio de Neurociencias, Instituto Nacional de Pediatría, Secretaría de Salud, Ciudad de México 04530, Mexico; america_vc@yahoo.com.mx; 5 Laboratorio de Parasitología Experimental, Instituto Nacional de Pediatría, Secretaría de Salud, Ciudad de México 04530, Mexico; yadirg@gmail.com; 6 Colegio de Ciencias y Humanidades, Plantel Casa Libertad, Universidad Autónoma de la Ciudad de México, Ciudad de México 09620, Mexico; edgar.sierra@uacm.edu.mx; 7 Departamento de Química de Biomacromoléculas, Instituto de Química, Universidad Nacional Autónoma de México, Circuito Exterior s/n, Ciudad Universitaria, Ciudad de México 04510, Mexico; e-rodriguez-bustamante@ciencias.unam.mx (E.R.-B.); arrespin@unam.mx (R.A.-E.)

**Keywords:** *Giardia lamblia*, stem-loop RT-qPCR, small nucleolar RNA, microRNA

## Abstract

Stem-loop quantitative reverse transcription PCR (RT-qPCR) is a molecular technique used for identification and quantification of individual small RNAs in cells. In this work, we used a Universal ProbeLibrary (UPL)-based design to detect—in a rapid, sensitive, specific, and reproducible way—the small nucleolar RNA (snoRNA) GlsR17 and its derived miRNA (miR2) of *Giardia lamblia* using a stem-loop RT-qPCR approach. Both small RNAs could be isolated from both total RNA and small RNA samples. Identification of the two small RNAs was carried out by sequencing the PCR-amplified small RNA products upon ligation into the pJET1.2/blunt vector. GlsR17 is constitutively expressed during the 72 h cultures of trophozoites, while the mature miR2 is present in 2-fold higher abundance during the first 48 h than at 72 h. Because it has been suggested that miRNAs in *G*. *lamblia* have an important role in the regulation of gene expression, the use of the stem-loop RT-qPCR method could be valuable for the study of miRNAs of *G*. *lamblia*. This methodology will be a powerful tool for studying gene regulation in *G. lamblia*, and will help to better understand the features and functions of these regulatory molecules and how they work within the RNA interference (RNAi) pathway in *G. lamblia*.

## 1. Introduction

*Giardia lamblia* is a flagellate protozoan that adapted over time to a parasitic lifestyle [1]. This organism causes the disease known as giardiasis in humans, but can also affect several vertebrate organisms [2]. Since *G. lamblia* is an early divergent microorganism, it has been taken as a good model to study gene regulation, because it shares mechanisms with both prokaryotic and eukaryotic organisms [3].

It is known that small molecules of RNA regulate important cellular processes in different eukaryotic organisms, including *G*. *lamblia* [4]. Small nucleolar RNA (snoRNA) has been identified as a source of microRNAs (miRNAs) in some protozoans and metazoans [5,6,7,8,9,10]. These miRNAs are small RNA molecules that regulate gene expression in lower and higher eukaryotes, but they have not been studied in detail in *Giardia* [11], although the origins and evolution of miRNAs and other regulatory small RNAs have been extensively characterized [12]. Some miRNAs (miR1, miR2, miR3, miR6, miR10) derived from snoRNA have been identified in *G*. *lamblia* by small RNA isolation and ligation to RNA linkers [6], and also by sequencing analysis followed by primer extension [7]. The biogenesis of the different classes of small RNAs shows important differences, as has been previously reported [5,13,14,15,16]. At least 166 miRNAs have been reported in *G*. *lamblia* founded by in silico prediction, analysis of homology searching, or deep sequencing [4,6,7,17,18]. Studies on the functionality of miRNAs indicated that they may have an important role on the regulation of genes encoding variant-specific surface proteins (VSPs) [6,7]; each of these miRNAs showed specific binding sites in the sequence of VSP genes [7,17,18].

Recently, it was reported that VSP genes are transcribed simultaneously in sense and antisense strands, which was proposed to result in the formation of double-stranded RNAs (dsRNAs) that are then degraded by the Dicer endonuclease complex [19]. Long dsRNAs can serve as templates for the biogenesis of small RNAs; this form of post-transcriptional gene silencing (PTGS) is a mechanism similar to the RNA interference (RNAi) pathway [20,21,22]. Other molecules, such as endo-small interfering RNAs (endo-siRNAs) and tRNAs derived from small RNAs, have been shown to play a key role in the differentiation of *Giardia*, suggesting that they might be involved in the regulation of this process [23]. These studies confirm that *G*. *lamblia* possesses expression regulatory mechanisms by PTGS or through the RNAi pathway involving small RNAs [6,18,21,22,23]. Moreover, several elements of the RNAi silencing pathway have been identified in *Giardia*, making conceivable its existence and usefulness to perform specific gene silencing. Analysis of the *Giardia* genome has revealed the presence of one copy each of the *Dicer*, Argonaute (*Ago*), and RNA-dependent RNA polymerase (*RdRp*) genes [6,21,24]. Although Drosha and Exportin 5 homologs are absent in this parasite, there is the possibility of another Drosha-independent pathway involved in miRNA biogenesis from snoRNA [4,6].

Because of the importance of small RNAs, different techniques have been used to detect miRNAs, including isolation of small RNA and ligation to RNA linkers (which requires the presence of a 5′-phosphate in the small RNA) [25], Northern blot [26,27], primer extension [28], microarrays [29], and real-time PCR [30]. In *G*. *lamblia*, fluorescence in situ hybridization (FISH) was used to find the cellular location of the snoRNA GlsR17 and its product, miR2 [6]; however, these techniques require time, and they can be laborious, expensive, and have low sensitivity [27]. Stem-loop quantitative reverse transcription PCR (RT-qPCR) is an efficient and novel strategy that has been used in recent years to detect and amplify mature miRNAs [31,32,33,34]. The stem-loop is designed such that it is shaped as a hairpin and possesses a 3′ overhang complementary to the miRNA. Also, a miRNA-specific forward primers with a 5′ adapter and a universal reverse primer are design for PCR amplification; these features allow a high specificity and increased flexibility in the design of primers for identification of microRNAs [35,36].

In this paper, we describe the use of the stem-loop RT-qPCR technique to detect different small RNAs in *G. lamblia*. With this technique, we could isolate, identify, and quantify the expression of GlsR17 (ID: AY820298.1) and its derived mature miRNA, miR2. As far as we know, this is the first report that demonstrates the use of the stem-loop RT-qPCR method for the identification and validation of small RNAs in this parasite. This technique can thus be adopted for the quantification of small RNAs in *G*. *lamblia*, and used in analyses of genes that are regulated by small RNAs through the RNAi pathway.

## 2. Materials and Methods

### 2.1. Strains and Experimental Conditions

The WB strain of *G. lamblia* was obtained from the American Type Culture Collection (ATCC). Trophozoites were grown in tubes containing 7 mL of TYI-S-33 medium (pH 7.02) supplemented with 10% fetal bovine serum and antibiotics (ampicillin, cephalothin, amphotericin, 10, 10 and 5 µg/mL, respectively) and incubated at 37 °C. Upon reaching a confluent monolayer (after approximately 60 h) the cells were placed on ice for 20 min and then collected by centrifugation at 3400× *g*; the medium was discarded completely and the cells were washed twice with phosphate saline buffer before RNA extraction.

### 2.2. RNA Samples: Total RNA and Small RNA

Total RNA was extracted using TRIzol^®^ Reagent (Invitrogen, Carlsbad, CA, USA) according to the manufacturer’s instructions. The concentration and the purity of the isolated RNA were quantified using a NanoDrop ND-1000 Micro-Volume (NanoDrop Technologies, Wilmington, DE, USA), and its integrity was verified in a 0.8% (*w*/*v*) agarose gel under denaturing conditions.

The extraction of small RNAs was performed with the mirPremier^®^ microRNA Isolation Kit (Sigma-Aldrich, St. Louis, MO, USA), following the instructions of the manufacturer’s protocol. The small RNA obtained was electrophoresed in 3% (*w*/*v*) agarose gels and visualized with GelRed (Nucleic Acid Gel, Biotium; Hayward, CA, USA) on a MultiDoc-It (UVP; Upland, CA, USA).

In addition, RNAs with sizes ≈100 bp were copurified from total RNA and obtained by the aforementioned kit, following the protocol of Malone et al. [37] and Nielsen [38], with some modifications for this work.

### 2.3. Primers and Endpoint PCR

The sequence for primer design of the GlsR17-Fw primer was obtained from GenBank, Accession ID: AY820298.1, and then compared to the reported sequence in the *Giardia* genome database (http://giardiadb.org/giardiadb/) [39]. The primers used in this study are shown in Table 1.

Primers were tested by endpoint PCR to analyze their specificity using cDNA as a template (see next section) and the enzyme Phusion High-Fidelity DNA polymerase (Thermo Scientific, Carlsbad, CA, USA), with the following amplification conditions: 30 s at 98 °C; 30 cycles of 10 s at 98 °C, 15 s at 60 °C, and 15 s at 72 °C; and, finally, 2 min at 72 °C. After amplification, the PCR products were separated by electrophoresis on a 3% (*w*/*v*) agarose gel. PCR products were also resolved by electrophoresis through 16% polyacrylamide gels to improve the resolution of the bands; these gels were performed according to the method reported by other authors [40], with some modifications. The gels were stained with GelRed (Nucleic Acid Gel, Biotium) and visualized on a MultiDoc-It (UVP). Synthesis of primers and sequence analysis were carried out at the Unidad de síntesis y secuenciación del Instituto de Biotecnología, UNAM (Cuernavaca, Mexico).

The PCR products were purified and ligated into the pJET1.2/blunt vector (CloneJET PCR Cloning Kit; Thermo Scientific). Each construction was transformed into competent *Escherichia coli* TOP10F´ cells (Invitrogen), which were grown at 37 °C overnight on Luria-Bertani (LB) agar plates supplemented with 100 μg/mL ampicillin. The plasmidic DNA was extracted by using the GeneJET Plasmid Miniprep Kit (Thermo Scientific), as indicated by the manufacturer, and then the cloned fragments were sequenced.

### 2.4. Stem-Loop RT-qPCR Assay Design

**Figure 1:**
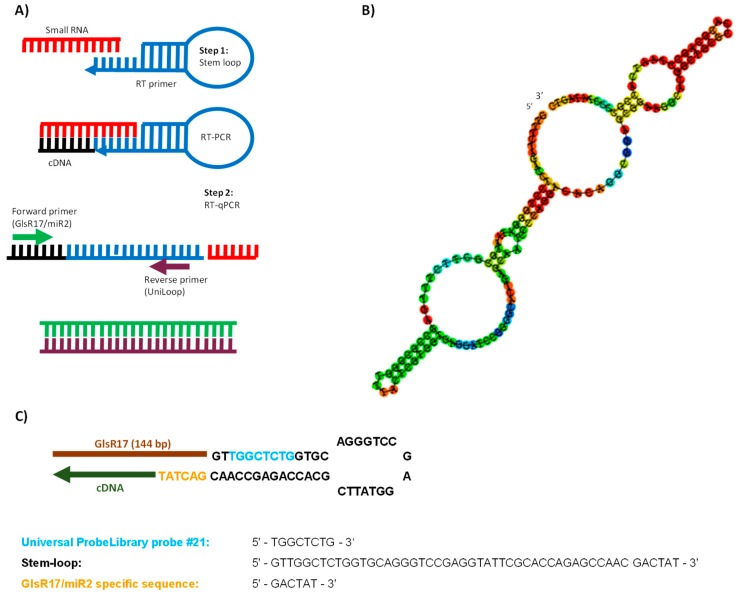
Stem-loop quantitative reverse transcription PCR (RT-qPCR) design to identify small RNAs of *Giardia lamblia* and schematic representation of the GlsR17 folding. (**A**) The scheme indicates the formation of complementary DNA (cDNA) from stem-loop-small RNA (step 1) and the quantification of the expression by RT-qPCR (step 2); (**B**) predicted structure of the small nucleolar RNA (snoRNA) GlsR17 based on RNAFold; (**C**) structure of the Stem-loop-GlsR17 probe hybridizing to the target small RNA; the cDNA generated by reverse transcription is shown in green color, the Universal ProbeLibrary (UPL) 21 region in blue, and the sequence specific for capturing the GlsR17 and miR2 RNAs (GACTAT) in yellow.

We are proposing a stem-loop RT-qPCR approach for the identification and quantification of small RNAs (snoRNA/miRNA) in *G*. *lamblia*. This methodology consists of two steps: reverse transcription (RT) and real-time PCR. First, the stem-loop probe Stem-loop-GlsR17 was used to specifically capture the snoRNA GlsR17 and perform reverse transcription with the Revertaid reverse transcriptase (Thermo Scientific). Then, the RT product was quantified by RT-qPCR using SYBR Green (Figure 1A). The Stem-loop-GlsR17 Universal ProbeLibrary (UPL) probe, the GlsR17- and miR2-specific primers, and the universal reverse primer (UniLoop) were all designed using the UPL probe-based stem-loop quantitative PCR assay design software (http://genomics.dote.hu:8080/mirnadesigntool) [32]. This software is open source under the GNU General Public License, and access to the source code can be requested by contacting the corresponding author of this software.

### 2.5. Synthesis of First-Strand cDNA

For complementary DNA (cDNA) synthesis, 1 µg of total RNA was first treated with the enzyme DNase I (Thermo Scientific) to remove DNA contamination. Complementary DNA was synthesized from DNase I-treated total RNA or from small RNA, using the Stem-loop-GlsR17 primer in 20 µL reaction volume with dNTP mix (10 mM) and Revertaid reverse transcriptase (Thermo Scientific). The reaction was incubated for 30 min at 16 °C followed by 60 min at 42 °C, and then stopped by heating at 70 °C for 10 min. The cDNA was stored at −20 °C until use.

### 2.6. RT-qPCR Assay

Amplification was carried out in a StepOne^TM^ Real-Time PCR System (Applied Biosystems, Foster City, CA, USA). The reactions were performed with the Fast SYBR^®^ Green Master Mix kit (Applied Biosystems), using the following conditions: 95 °C for 30 s, followed by 40 cycles of 95 °C for 3 s and 60 °C for 30 s. Upon completion of the reaction cycles, melt curves were obtained by heating the reactions from 60 °C to 95 °C. The specificity of the primers was confirmed by the presence of a single peak in the melt curve generated for GlsR17 and miR2. Negative controls were a reaction lacking the reactive and a reaction without template (NTC control) to verify the absence of contaminating DNA. Calculation of the PCR amplification efficiency for each primer was performed using 10-fold dilution series of cDNA as a template and applying the formula E = 10^(−1/k)^ [41,42,43], where k is the slope. The slope value was obtained from the standard curve generated by the StepOne software v2.3 (Applied Biosystems). Normalization was performed using the geometric mean values of the cycle thresholds (Ct). Finally, the 2^−ΔΔCt^ method was used for the quantification of the relative expression of the small RNAs of interest [44]. The expression level of the aldolase (*Ald*) housekeeping gene was employed for equal loading control in all experiments [45]. Five replicates were made of each reaction for small RNAs. The results are expressed as values of the mean ± standard deviation (SD). Data analysis was performed using the software StepOne, version 2.3 (Applied Biosystems).

## 3. Results

### 3.1. Strategy for Detecting Small RNAs of G. lamblia

The most important consideration for designing a strategy to specifically detect a specific small RNA is to know its precise sequence. *G. lamblia* possesses snoRNAs, some of which have been proven to be precursors of mature miRNAs involved in the regulation of several genes [6,7]. We chose the snoRNA GlsR17 (ID: AY820298.1) and its derived miRNA (miR2) to design a stem-loop RT-qPCR-based strategy that could be routinely applied to detect and quantify small RNAs in *G. lamblia*.

The sequences corresponding to miR2 are located at the 3′-end of GlsR17 (Figure 1B) and a D box (CUAA) is present in the second hairpin structure of *G. lamblia* GlsR17, within the miR2 sequences, which identifies GlsR17 as a snoRNA of the C/D box class [46]. The relatively small size and the hairpin loop structures of GlsR17 qualify it as suitable substrate for Dicer action [5]. The sequence of the specific stem-loop-GlsR17 probe (based on the universal UPL21 probe) was designed with the software miRNA Primer Design Tool [32]. The stem-loop structure of the Stem-loop-GlsR17 probe hybridizing to the target small RNA is shown in Figure 1C; it contains the hexanucleotide GACTAT at the 3′-end, specific for capturing the GlsR17 and miR2 RNAs since they both possess the complementary AUAGUC sequence at their 3′-ends.

### 3.2. GlsR17 and miR2 Amplification by Stem-Loop RT-PCR

**Figure 2:**
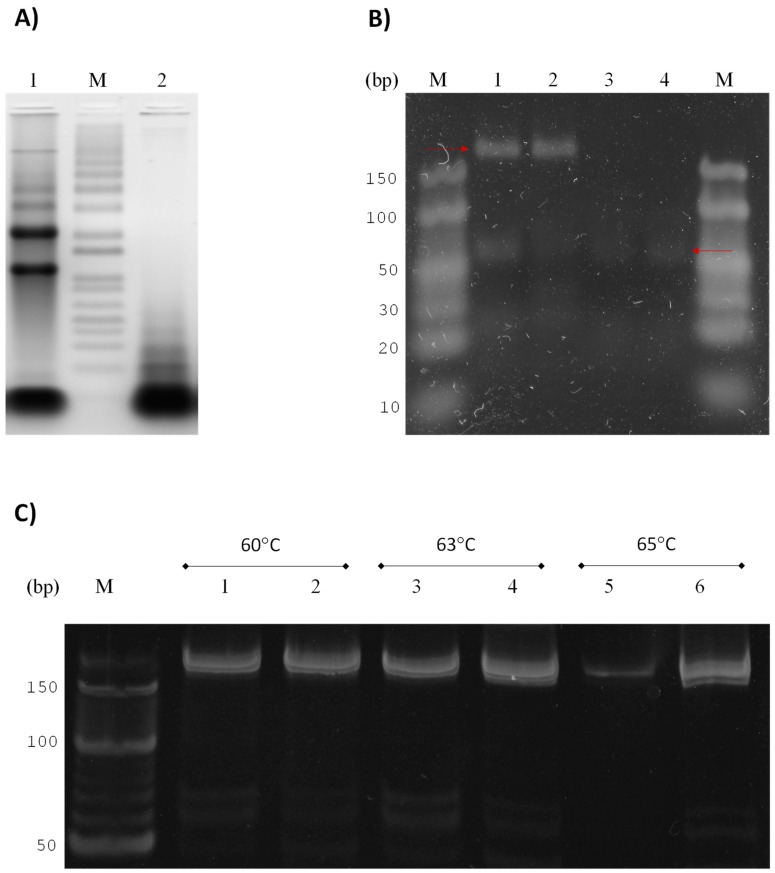
Identification of PCR-amplified fragments as GlsR17 and miR2 from *G*. *lamblia*. (**A**) Total RNA (lane 1) and small RNA (lane 3) extracted from *G*. *lamblia* trophozoites (see Materials and Methods) run on a 2% agarose gel; the size marker (M) was 1 kb (lane 2) (O’GeneRuler 1 kb DNA Ladder, Thermo Scientific); (**B**) agarose gels (3%) with the result of the amplification stem-loop RT-PCR analysis for GlsR17 and miR2 (lanes 1–4), respectively, using total RNA (lanes 1 and 3) or small RNA (lanes 2 and 4) as a template. The expected size for the GlsR17 amplified fragment was 178 bp, and for the miR2 fragment 61 bp, which is in accordance with the size of the bands appearing in the gels; (**C**) GlsR17 amplification from a temperature gradient by PCR and run on a 16% polyacrylamide gel. Products obtained from total RNA (lanes 1, 3 and 5) and small RNAs (lanes 2, 4 and 6). To determine the size of the fragments corresponding to the small RNAs the marker (M) O’RangeRuler 10 bp DNA Ladder (Thermo Scientific) was used.

The reported sizes for GlsR17 and miR2 are 144 and 25 nucleotides, respectively [6], so we used a sample of small RNA (see Materials and Methods) in addition to total RNA (Figure 2A) to perform the first-strand cDNA synthesis. According to the design of the primers for amplification by PCR, the expected sizes of the amplified fragments corresponding specifically to either GlsR17 or miR2 were ≈178 and 61 bp, respectively. The agarose gel electrophoresis of the PCR-amplified products showed bands of the expected size for both GlsR17 and miR2 (Figure 2B). As can be observed, the gels showed no difference in size or intensity between the PCR products obtained from total RNA and from small RNA, which validates the use of the more readily obtainable total RNA for detection of small RNAs by stem-loop RT-PCR in *G*. *lamblia*. Because the resolutions of the agarose gels were not suitable, we decided to work with polyacrylamide gels for better definition of bands.

Then, the amplicons obtained for the GlsR17 by conventional PCR using a temperature gradient were resolved in these gels; no differences were found in products amplified between 60 and 75 °C from samples of total RNA or small RNAs (Figure 2C).

### 3.3. Amplification of miR2 by PCR

**Figure 3:**
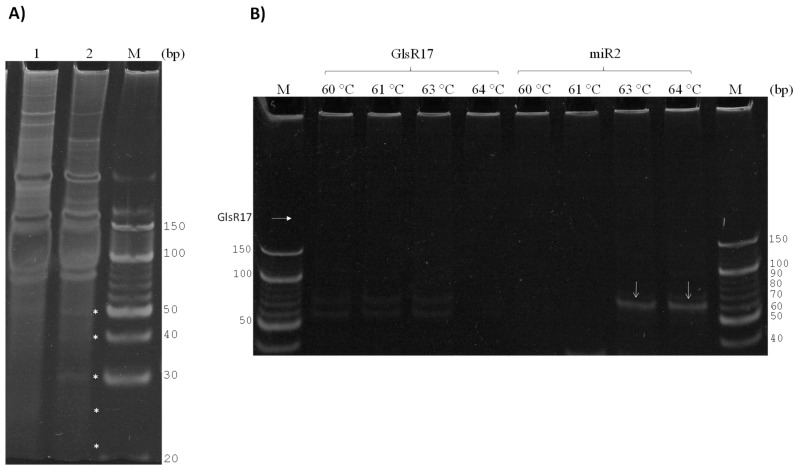
Size selection and gel extraction of small RNAs from total RNA and identification of PCR-amplified fragments of miR2. (**A**) Small RNAs obtained with sizes smaller than 100 bp copurified from total RNA (lane 1) and small RNA (lane 2), previously shown in Figure 2A. * Indicates bands of small RNAs between sizes 20 and 50 bp; (**B**) PCR was performed to verify the absence of GlsR17 and confirm the presence of miR2 in samples of small RNA less than 100 bp. Gradient of temperature was performed and the amplicons were observed on a 16% polyacrylamide gel.

Importantly, we demonstrated that from total RNA and small RNAs purified, both GlsR17 and miR2 were amplified. However, we needed to verify that the miRNA (miR2) obtained is not generated from the GlsR17 amplicon being used as template, and that it originated naturally by Dicer processing. For this reason, we decided to copurify small RNAs with sizes less than 100 bp (Figure 3A) from RNAs previously obtained (Figure 2A). When these small RNA fractions with less than 100 bp were used, amplicons of the snoRNA not were obtained despite using a temperature gradient between 60 and 64 °C (Figure 3B), while a 61 bp amplicon was obtained with the primers corresponding to the miRNA miR2 (Figure 3B), and the miR2 products were obtained only at 63 and 64 °C; this result led to the conclusion that the miR2 obtained was formed as a result of GlsR17 processing.

**Figure 4:**
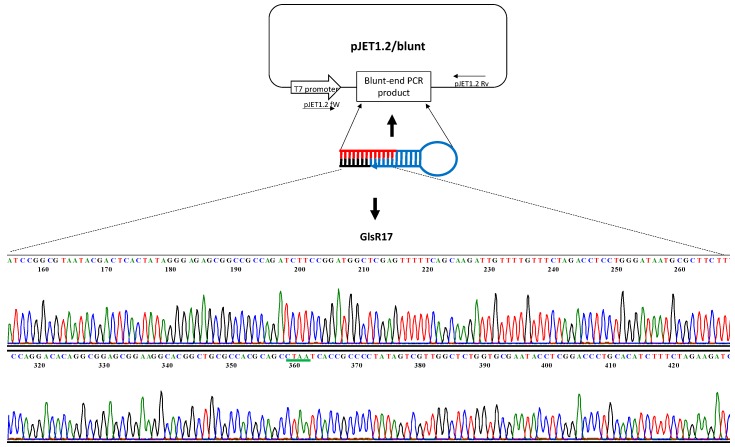
Identification of PCR-amplified fragments of GlsR17 from *G*. *lamblia*. PCR-amplified fragments with the primer pairs GlsR17-Fw and UniLoop were cloned into the pJET2.1/blunt vector and sequenced. The corresponding sequence identified the fragment of GlsR17. The green line indicates the sequences of the D box.

After this, the amplified fragments were ligated to the pJET2.1/blunt vector and sequenced. The resulting sequences showed the presence of a 144 bp fragment corresponding to GlsR17 (Figure 4) and the other sequence that corresponds to the cDNA synthesized from miR2, showing an identity of 100% with the sequence of AY820298 and L2708 reported in the NCBI BLASTN database. In both sequences, a D box sequence (CTAA) is present, which is a characteristic element of the snoRNA and miR2 of *G*. *lamblia*.

### 3.4. Validation of Primers for RT-qPCR

**Figure 5:**
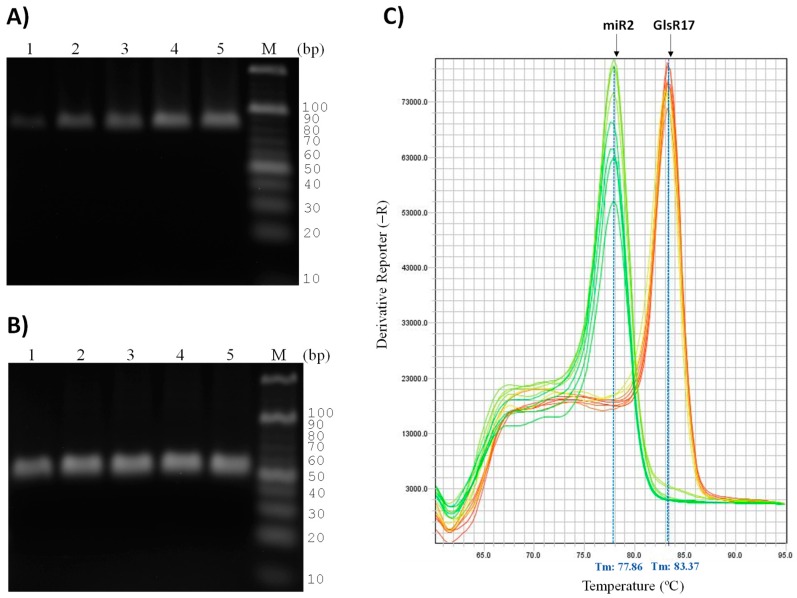
Amplification of the snoRNA GlsR17 and its derived miRNA (miR2) by PCR and RT-qPCR. Agarose gels (3%) with the PCR amplification products obtained with primers GslR17-Fw and UniLoop (**A**) and miR2-Fw and UniLoop (**B**) using different concentrations of template cDNA: lane 1: 0.025 ng, lane 2: 0.25 ng, lane 3: 2.5 ng, lane 4: 25 ng, lane 5: 250 ng. Lane M: O’RangeRuler^TM^ 10 bp DNA Ladder (Thermo Scientific). PCR conditions were: 95 °C for 30 s, and 40 cycles of 95 °C for 3 s and 60 °C for 30 s; (**C**) melt curves obtained for GlsR17 and miR2 amplification products by real-time PCR were generated using SYBR Green, and the same primers, template concentrations, and conditions as mentioned in A and B.

To set the conditions and validate the primers GlsR17-Fw, miR2-Fw, and UniLoop for RT-qPCR, we performed a series of PCR reactions using different amounts of cDNA, previously obtained with the probe Stem-loop-GlsR17, as a template (from 0.02 to 200 ng). As can be observed in Figure 5, amplification products of the expected size were obtained even with the smallest cDNA concentration for both GlsR17 (Figure 5A) and miR2 (Figure 5B). To test the specificity of the primers and confirm the results observed in the agarose gels, we performed RT-qPCR reactions in presence of SYBR Green and submitted the products to denaturation by increasing the temperature to obtain melt curves. A single well-defined sharp peak for each concentration was observed in both the GlsR17 and miR2 reactions (Figure 5C); the melting temperature (T_m_) for the amplified products, according to the melt curves, were 77.86 °C and 83.37 °C, respectively. This result confirms that the oligonucleotides used in the reaction are specific for each of the two small RNAs. The amplification efficiency was 98% for GlsR17 and 101% for miR2, which are adequate for relative quantification of expression by RT-qPCR using the 2^−ΔΔCt^ method [42,43,45,47].

### 3.5. Quantification of Expression of GlsR17 and miR2

**Figure 6:**
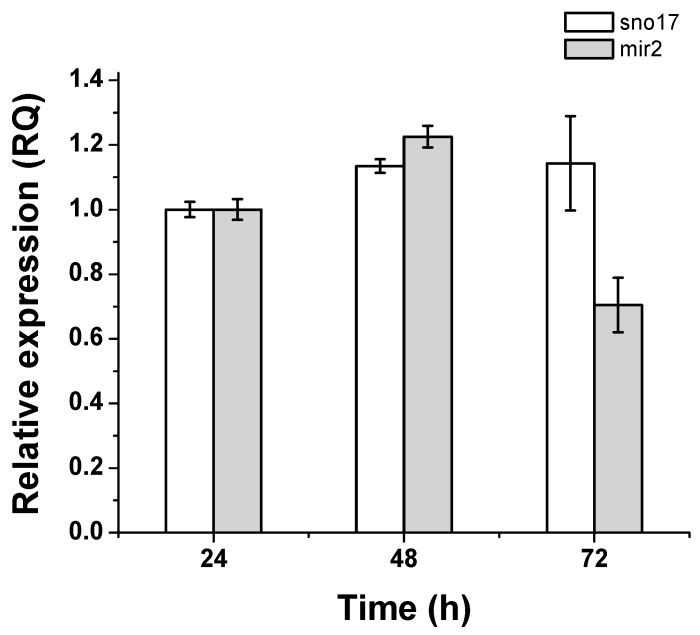
Quantification of the relative abundance of GlsR17 and miR2 from *G*. *lamblia.* The trophozoites were grown in TYI-S-33 medium for 72 h and the samples were taken every 24 h. The error bars indicate standard deviation (SD) from five replicates, see Materials and Methods for details.

We performed an analysis of the abundance of GlsR17 and miR2 during the first 72 h of a culture of trophozoites in TYI-S-33 medium, with samples taken every 24 h (Figure 6). The analysis was performed by RT-qPCR according to the conditions established in the previous section, with 20 ng of cDNA as a template. The results showed that from 24 to 72 h of culture, GlsR17 is present in similar abundance, with a slight increase from 24 to 48 h, whereas miR2 abundance peaks at 48 h and drops to half the amount at 72 h. The decrease of the miR2 expression could be mediated by changes of miRNAs expression in axenic culture, similar to the mRNA transcriptome changes of *Giardia duodenalis* trophozoites in these conditions for 96 h (analyzing stages of growth, stationary and declining), characterized by Ansell et al. [48]. Then, we consider that this change in the expression of miR2 is related to the expression of the transcriptome of *G*. *lamblia* in the time studied and with the regulation of different genes. Also, changes in the expression of VSP genes in *Giardia* have been reported, and could be related to the levels of the regulatory molecule, miR2. As previously demonstrated, miR2 is involved in regulating the expression of VSP genes, proteins that exhibit increase during stationary and decay stages (60–96 h) of *G*. *lamblia* trophozoite [6,48].

### 3.6. Identification of miR2 Targets in VSP Genes

**Figure 7:**
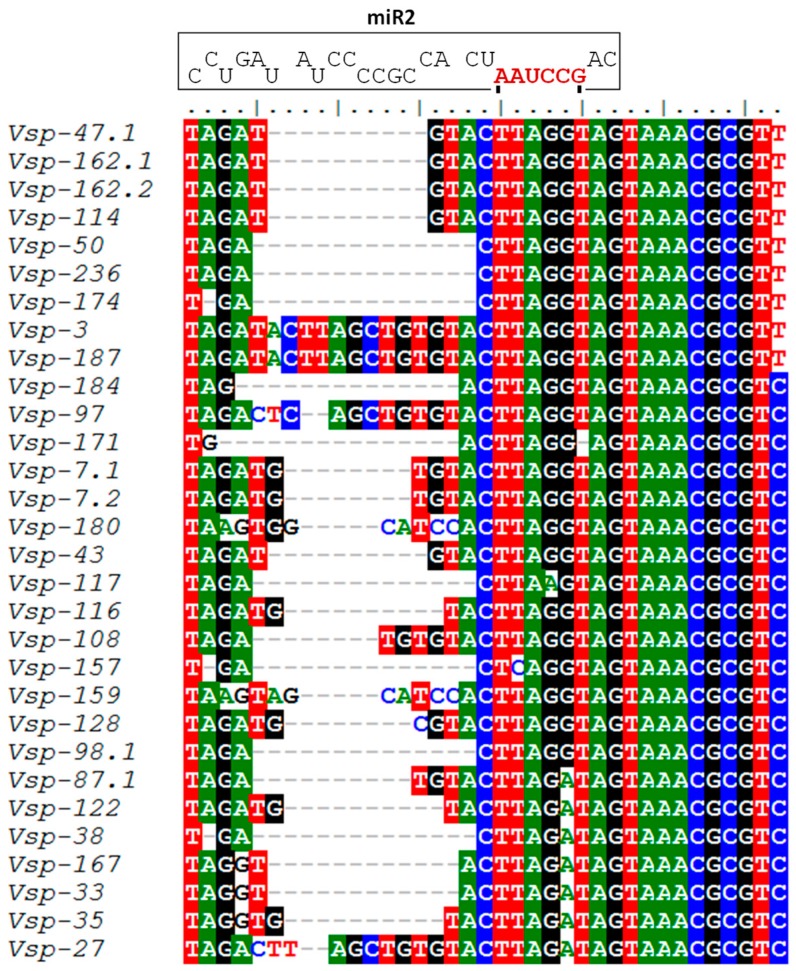
Alignment of different VSP genes. Conserved sequences and binding sites with the microRNA miR2 (brown color) in the 3′-untranslated region (3′-UTR) region of VSP genes from *G*. *lamblia*. *VSP-47.1* (GL50803_117203), *VSP-162.1* (GL50803_111936), *VSP-162.2* (GL50803_111933), *VSP-114* (GL50803_113304), *VSP-50* (GL50803_137710), *VSP-236* (GL50803_122564), *VSP-174* (GL50803_137604), *VSP-3* (GL50803_137740), *VSP-187* (GL50803_89315), *VSP-184* (GL50803_101380), *VSP-97* (GL50803_10562), *VSP-171* (GL50803_13402), *VSP-7.1* (GL50803_136003), *VSP-7.2* (GL50803_136004), *VSP-180* (GL50803_137614), *VSP-43* (GL50803_40591), *VSP-117* (GL50803_97820), *VSP-116* (GL50803_101765), *VSP-108* (GL50803_101765), *VSP-157* (GL50803_103916), *VSP-159* (GL50803_112113), *VSP-128* (GL50803_112207), *VSP-98.1* (GL50803_112208), *VSP-87.1* (GL50803_112647), *VSP-122* (GL50803_113357), *VSP-38* (GL50803_13194), *VSP-167* (GL50803_134710), VSP-33 (GL50803_134711), *VSP-25* (GL50803_37093), *VSP-27* (GL50803_38901). The colors represent the following: red: thymine (T); green: adenine (A); black: guanine (G); and blue: cytosine (C). The alignment was made with the bioinformatic program BioEdit (Ibis Biosciences, Carlsbad, CA, USA), current version 7.2.5.

The miRNA miR2 potentially participates in the regulation of at least 22 different genes encoding variant-specific surface proteins (VSPs), since putative miR2 binding sites are present at the 3′-untranslated regions (3′-UTR) of their mRNAs [6]. These authors also demonstrated the functionality of miR2 in translational repression of the reporter protein luciferase, expressed a genetically engineered gene that produces an mRNA containing miR2 binding sites at the 3′-UTR. Considering the importance of antigenic variation for successful *G. lamblia* infection and the role that miR2 seems to have in this phenomenon, we decided to perform a sequence alignment with a larger number of sequences of genes encoding VSP proteins. As shown in Figure 7, 33 VSP genes were found to have conserved sequences that may bind miR2.

## 4. Discussion

The study of small RNAs, including miRNAs, siRNAs, and PIWI-interacting RNA (piRNA), is of great importance due to their role in gene regulation in different organisms [13,49]. One of the limitations in the study of these small molecules is the sensitivity of the methods used to detect a specific molecule and to analyze its expression levels in cells [27,50,51]. One method widely used for miRNAs detection and quantification is Northern blotting [24]; however, this methodology takes time for processing and may have low sensitivity [27]. Another method used has been primer extension [28]. It is thought that microarrays could improve the performance of the expression profiles of miRNAs, nonetheless, the method remains limited in terms of sensitivity (difficult to amplify the small RNA targets) and specificity (may lead to false positive signal) [29,52,53]. RT-qPCR has become the gold standard test for quantifying gene expression, however, applying it to miRNA or other small RNAs is a challenge since this technique requires a template that is at least twice as long as the specific forward or reverse primers, each typically ≈20 nt in length. Consequently, the target minimum length has to be around 40 bp, which makes most mature miRNAs and siRNAs too short for detection and quantification by standard RT-qPCR methods [30,53,54]. Another technique that could be adapted for the identification of small RNA is loop-mediated isothermal amplification (LAMP), which is a novel nucleic acid amplification method. Amplification and detection of amplicons can be completed in a single step, by incubating all the reagents in a single tube [55]. However, the LAMP reaction requires a set specially designed primers, uses four (or six) primers targeting six (or eight) regions with significant restrictions imposed on their respective positioning and orientation [55,56]. Another limitation for this technique, as well as those previously mentioned, is that they are not useful for cloning, and, therefore, it is not possible to get the sequence of the obtained amplicons.

Therefore, it is of utmost interest to have techniques available that can reliably detect and quantify small RNAs. In this sense, the stem-loop RT-qPCR technique has proven to be highly specific to detect and suitable to quantify specific small RNAs [31,32,33,36,53,57]. Moreover, this technique is fast and easy to perform and has a lower cost compared with Northern blotting. In this work, we have established the conditions for routine analysis of small RNAs in *Giardia*: the RNA extraction method, the design of the stem-loop probe and PCR primers, and the conditions of RT-qPCR to quantify the expression of specific small RNAs, using the snoRNA GlsR17 and its derived miRNA product (miR2) as isolation and quantification tests.

snoRNAs are essential for the maturation of ribosomal RNA, and are localized to the nucleolus [5,46]. Some snoRNAs also serve as precursors for the biogenesis of mature miRNAs that participate in gene regulation [5,9,10,58]. This is the case for some snoRNAs of *G. lamblia* [6,7]. Previous studies of miRNAs in *G*. *lamblia* were performed by small RNA isolation and ligation to RNA linkers [6], construction of a small RNA library in a plasmid vector [17], and bioinformatic sequence analysis followed by primer extension [7]. The miRNA miR2 was identified as a Dicer-processed product of the snoRNA GlsR17 and localized to the cytoplasm by FISH, whereas GlsR17 was found primarily in the nucleolus of only one of the two nuclei in *Giardia* [6]. The feasibility to detect and quantify specific miRNAs and other small regulatory RNAs by stem-loop RT-qPCR in *G. lamblia* will greatly contribute to the study of gene regulation and the role of miRNA in the possible antigenic variation in this parasite.

*G*. *lamblia* infections are associated with antigenic variation, which is generated by a continuous change of the VSPs expressed. From a repertory of around 190 VSP-encoding genes, *Giardia* can express only one VSP on the surface of each parasite at a particular time, and it spontaneously changes to a different VSP by unknown mechanisms [21]. The differential expression of GlsR17 and miR2 that we found in this study may be related to the timing of expression of VSP genes [48]. Recently, an analysis performed on the transcriptome of *G*. *duodenalis* showed transcriptional variation in axenic culture, during 96 hours, which includes the log, stationary, and declining stages of the in vitro culture [48]. As with this differential gene expression, miRNAs may also change their expression, as was observed with miR2 in this study (Figure 6). Although, we now observe that *VSP-116* (GL50803_101765) is one of the genes expressed mostly after 60 h of culture, and according to the alignment performed for these genes and their miR2 binding sites, this gene or others could be regulated by this miRNA (Figure 7). In addition, the increase in *VSP-116* gene expression may be due to the fact that miR2 negatively regulates its expression, among other genes, and these changes may be necessary for the survival of *G*. *lamblia*. This possible regulation by microRNA in *G. lamblia*, from which a bioinformatic analysis was conducted predicting different microRNAs derived from snoRNAs, and putative target sites were identified at the 3′-UTR of variant surface protein mRNAs.

The results reported by these authors confirmed that each of the miRNAs represses the translation of a reporter (luciferase gene) transcript tagged with their respective target sites, and this repression requires the presence of the Ago protein from *Giardia*, suggesting that it is supported by an RNA-induced silencing complex (RISC)-mediated mechanism [6,17,18].

## 5. Conclusions

In summary, our results demonstrate the feasibility of using the stem-loop RT-qPCR method for routinely and efficiently detecting and quantifying specific small RNAs (snoRNA/dsRNA/siRNA/miRNA) from total RNA in *G*. *lamblia*. Interestingly, we only used three primers to identify two small RNAs, one of them with a stem-loop to capture both small RNAs (GlsRNA17 and miR2), another that helped to obtain a specific small RNA by RT-PCR, and a third primer present in the internal sequence of the stem-loop (UniLoop-rev), which served to confirm by conventional PCR the capture of some small RNA. This will contribute to the study of gene regulation in *G. lamblia*, and in particular, to the role of miRNAs in antigenic variation through silencing of the expression of VSP proteins.

## Figures and Tables

**Table 1:** Primers used in this study.

Primer	Sequence (5′–3′)
Stem-loop-GlsR17	GTTGGCTCTGGTGCAGGGTCCGAGGTATTCGCACCAGAGCCAACGACTAT
GlsR17-Forward	TGTTTTGTTTCTAGACCTCCTGG
qGlsR17-Forward	CAGGACACAGGCGGAG
miR2-Forward	TGCAGCCTAATCACCGC
UniLoop-Reverse	GTGCAGGGTCCGAGGT
pJET1.2 Forward	CGACTCACTATAGGGAGAGCGGC
pJET1.2 Reverse	AAGAACATCGATTTTCCATGGCAG
pJET2-Forward	CAATTAGTAGCATCACGC
Ald-Forward	GAGTCCGTGAAGATGGCGA
Ald-Reverse	GTCCCAAGTTCAGCCTCCAC
